# Predicting Future Pandemics and Formulating Prevention Strategies: The Role of ChatGPT

**DOI:** 10.7759/cureus.44825

**Published:** 2023-09-07

**Authors:** Pradip K Jana, Agniva Majumdar, Shanta Dutta

**Affiliations:** 1 Regional Virus Research and Diagnostic Laboratory, Indian Council of Medical Research, National Institute of Cholera and Enteric Diseases, Kolkata, IND; 2 Bacteriology, Indian Council of Medical Research, National Institute of Cholera and Enteric Diseases, Kolkata, IND

**Keywords:** artificial intelligence, pandemic, outbreak, machine learning, chatbot, chatgpt, ai & robotics in healthcare

## Abstract

The emergence of severe acute respiratory syndrome coronavirus 2 (SARS-CoV-2) in late 2019 and its subsequent worldwide spread has emphasized the urgent need for better approaches for predicting and managing infectious disease outbreaks. One potential instrument in this effort is artificial intelligence (AI), and in specific, language models like ChatGPT (Chat Generative Pre-trained Transformer). In the present study, to explore how ChatGPT could predict future pandemics and give suggestions about the prevention strategy, our research team chatted with ChatGPT with several questions on July 12, 2023. Based on our conversation, we can conclude that AI is not a substitute for human expertise, but an adjunct to support early prediction, prevention, and management of future pandemics.

## Editorial

Since the World Health Organization announced that coronavirus disease 2019 (COVID-19) is no longer a public health emergency of international concern [1], a key question has started arising: Which organism will most likely cause the next pandemic? Throughout the centuries, the world has been afflicted by a range of infectious diseases such as the bubonic plague, Spanish flu, severe acute respiratory syndrome (SARS), Middle East respiratory syndrome (MERS), Ebola, Nipah, and more recently, COVID-19. The rapid globalization and increased travel across continents in modern times have further amplified the capacity of these diseases to evolve as pandemics, creating a persistent threat to human survival. The golden lesson we learned from the recent COVID-19 pandemic is that prevention beats cure. Despite substantial progress in scientific and technological advancements, the COVID-19 pandemic faced various challenges, such as a lack of sufficient prior knowledge of biological threats and public health preparedness. In this context, an innovative approach for predicting future pandemics is the key factor in boosting efficiency and preparedness for future encounters. Predicting future events with conviction is beyond our grip. However, by considering past outbreaks, analyzing large datasets, and assessing multiple factors and variables, we can gather hints about what pandemic may lie ahead. The first attempt to predict the spread of disease was made in the 14th century during the historical Black Death epidemic in Europe [2]. In 1850, during a cholera outbreak in London, John Snow analyzed the available data and proposed that certain areas served by a particular water pump were more affected than others [3]. Over the last few months, researchers explored the utility of machine learning approaches to predict the COVID-19 outbreak. Sina F. Ardabili et al. deployed a machine learning model to predict the COVID-19 outbreak as a substitute for susceptible-exposed-infectious-removed (SEIR) and susceptible-infected-recovered (SIR) models and they suggested machine learning as an effective tool for long-term prediction [4]. Another study by Gergo Pinter et al. demonstrated the potential of a hybrid machine-learning approach for predicting the COVID-19 pandemic in Hungary [5]. Based on the coronavirus's whole genome sequence, deep learning models have been fruitfully used in another study to predict the likelihood of cross-species infection for early pandemic warnings [6]. Investigators also explored the capability of ChatGPT (Chat Generative Pre-trained Transformer; OpenAI, San Francisco, CA) in predicting common drug-drug interactions [7], and upcoming technology on diabetes [8]. To the best of our knowledge, there is no existing literature examining the capability of this artificial intelligence (AI) tool to predict future pandemics. In this present manuscript, we will explore the role of ChatGPT in predicting future pandemics and its potential to improve our capability to manage upcoming encounters.

In the era of AI, ChatGPT is currently in a boom and already is used by millions of users. This natural language processing (NLP) model was developed by the San Francisco-based AI research and deployment organization OpenAI and released in November 2022 [9]. Its popularity has been mostly attributed to its capacity to engage in natural language conversations. The emergence of a novel organism, alternations of environmental conditions, host-pathogen interaction, and worldwide travel patterns are the various factors that directly or indirectly influence our predictive proficiencies regarding a pandemic. In this context, ChatGPT can enhance our predictive capabilities by evaluating epidemiological, environmental, clinical, news article, and social media datasets, as well as satellite imagery, health surveillance, and genomic sequencing data. Examining the data on the genetic makeup of pathogens and relating them to already existing treatments and vaccines, this AI tool can accelerate the development of potential treatments and vaccines for new pathogens. Our research team talked with ChatGPT on July 12, 2023, to explore how it might predict the future pandemic and give suggestions on prevention strategies from the AI dimensions. The entire conversation is described in Table 1.

**Table 1:** Responses from the ChatGPT language model for the future pandemic and prevention strategy-related questions.

Prompts	Response from ChatGPT
Prompt 1: Who are you?	Response 1: I am ChatGPT, an AI language model developed by OpenAI. I'm designed to assist with answering questions, providing information, and engaging in conversation on a wide range of topics. How can I help you today?
Prompt 2: Do you have specialized knowledge and training in the area of infectious diseases?	Response 2: As an AI language model, I have been trained on a wide variety of topics, including infectious diseases. I have access to a vast amount of information on the subject and can provide general knowledge and explanations. However, it's important to note that my responses are generated based on patterns and examples in the training data I've been exposed to, rather than personal experience or specialized training. While I strive to provide accurate and up-to-date information, it's always a good idea to consult authoritative sources and medical professionals for specific or current information related to infectious diseases.
Prompt 3: What are the possible organisms that may lead to the next pandemic after COVID-19?	Response 3: Predicting the exact organism that could lead to the next pandemic is challenging, as it depends on various factors such as viral evolution, zoonotic transmission, and human behavior. However, several types of organisms have the potential to cause future pandemics. Here are some examples: Coronaviruses: Coronaviruses, like the one responsible for COVID-19, are a family of viruses that can cause respiratory illnesses in humans. They have the potential to undergo genetic mutations and cross the species barrier, leading to the emergence of novel strains. Influenza viruses: Influenza viruses have caused previous pandemics, such as the H1N1 influenza pandemic in 2009. They continue to evolve, and new strains with pandemic potential could emerge in the future. Filoviruses: Filoviruses, including Ebola and Marburg viruses, have caused outbreaks with high mortality rates. While these viruses primarily circulate in specific regions of Africa, there is a potential risk of them spreading to other regions. Paramyxoviruses: Paramyxoviruses, such as Nipah and Hendra viruses, are zoonotic viruses that have caused outbreaks in the past. They have the potential to cause severe respiratory illnesses in humans. Bacterial pathogens: While viral infections often come to mind when discussing pandemics, bacterial pathogens can also pose a significant risk. Examples include antibiotic-resistant bacteria, such as methicillin-resistant Staphylococcus aureus (MRSA) and multidrug-resistant tuberculosis (MDR-TB). It's important to note that this list is not exhaustive, and there are other organisms that could potentially lead to future pandemics. Ongoing surveillance, research, and preparedness are crucial in monitoring and mitigating the risks associated with emerging infectious diseases.
Prompt 4: Do you want to add more organisms to the list?	Response 4: Certainly! Here are a few more organisms that could potentially lead to future pandemics: Retroviruses: Retroviruses, such as HIV, are known for their ability to undergo genetic mutations and adapt to new hosts. Although significant progress has been made in managing HIV/AIDS, new strains or recombinant forms could pose challenges in the future. Arboviruses: Arboviruses, transmitted by arthropods like mosquitoes and ticks, have the potential to cause widespread outbreaks. Examples include dengue fever, Zika virus, West Nile virus, and chikungunya virus. Fungal pathogens: While less commonly associated with pandemics, certain fungal pathogens can cause severe infections, particularly in individuals with weakened immune systems. Candida auris, for instance, has emerged as a multidrug-resistant fungal pathogen that poses a significant threat in healthcare settings. Prion diseases: Prion diseases are rare, transmissible neurodegenerative disorders caused by abnormal proteins. Examples include Creutzfeldt-Jakob disease (CJD) and variant CJD (vCJD). Although not easily transmissible, the potential for prion diseases to become more contagious is an area of ongoing research. Unknown or novel pathogens: It's important to recognize that new and unknown pathogens could emerge and lead to pandemics. The ongoing surveillance of wildlife, early detection systems, and rapid response capabilities are critical in identifying and managing such novel threats. These examples demonstrate the diverse range of organisms that could potentially contribute to future pandemics. Continued research, surveillance, and preparedness efforts are crucial in identifying and mitigating these risks to global health.
Prompt 5: Can you explain the different stages involved in the emergence of an infectious disease?	Response 5: The emergence of an infectious disease typically involves several stages, which can vary depending on the specific pathogen and the circumstances surrounding its transmission. Here are the general stages involved in the emergence of an infectious disease: Spillover event: The initial stage involves the spillover event, where a pathogen is transmitted from its natural reservoir host (often an animal) to a new host species, such as humans. This can occur through various mechanisms, including direct contact with infected animals, consumption of contaminated food, or exposure to environmental sources. Localized outbreak: After the spillover event, the pathogen may cause a localized outbreak within the new host population. This could be limited to a small geographical area or a specific group of individuals. The outbreak may initially go unnoticed or be misdiagnosed as common illnesses, making it challenging to identify. Transmission and spread: If the pathogen has the ability to transmit efficiently between individuals, it can lead to sustained human-to-human transmission. This stage is crucial for the pathogen's potential to cause a larger-scale epidemic or pandemic. Factors influencing transmission include the mode of transmission (e.g., respiratory droplets, contaminated surfaces), the pathogen's infectiousness, and the susceptibility of the population. Epidemic or pandemic: In this stage, the disease spreads extensively within a population, leading to a significant increase in cases. An epidemic refers to the rapid and widespread occurrence of cases within a defined region, while a pandemic refers to a global epidemic, affecting multiple countries or continents. Public health response: As the disease outbreak progresses, public health authorities and healthcare systems respond to control the spread and mitigate the impact. Measures may include increased surveillance, contact tracing, isolation and quarantine, travel restrictions, public awareness campaigns, development and distribution of vaccines or treatments, and other interventions aimed at reducing transmission. Resolution or endemicity: Depending on the effectiveness of control measures and the characteristics of the pathogen, the outbreak may eventually be brought under control, leading to a decline in cases. In some cases, the disease may become endemic, meaning it persists at a relatively stable level within a particular geographic area or population. It's important to note that these stages are generalized and may not apply to every infectious disease outbreak. Each pathogen and outbreak situation can have unique characteristics, requiring tailored approaches in terms of prevention, surveillance, and response strategies.
Prompt 6: What are the best stages to intervene with a prevention strategy to prevent the emergence of a disease?	Response 6: Intervening at different stages can be important in preventing the emergence of a disease. Here are key stages where prevention strategies can be effective: Pre-spillover stage: The pre-spillover stage involves identifying and understanding potential zoonotic pathogens in their natural reservoirs. Surveillance systems can be established to monitor and study animal populations that harbor pathogens with zoonotic potential. This early detection and characterization can aid in assessing the risk and informing preventive measures. Spillover event stage: Intervening at the spillover event stage is crucial. Measures such as monitoring high-risk areas, identifying potential interfaces between humans and animals (e.g., wildlife markets, farms), and promoting awareness of zoonotic diseases can help minimize the chances of spillover. Enhanced hygiene practices, safe handling and processing of animals, and improved animal health management can reduce the risk of transmission. Early outbreak stage: Detecting and responding to localized outbreaks early can prevent further transmission and larger-scale epidemics. This involves improving surveillance systems to rapidly identify and diagnose emerging diseases, especially in areas where outbreaks are likely to occur. Robust laboratory capacity, trained healthcare workers, and effective reporting mechanisms are essential for timely detection and response. Transmission control stage: Implementing preventive measures during the transmission and spread stage is critical for minimizing the impact of an outbreak. This includes promoting infection prevention and control practices (e.g., hand hygiene, respiratory etiquette), implementing targeted vaccination campaigns if available, promoting appropriate use of antimicrobials to reduce resistance, and ensuring access to healthcare for early diagnosis and treatment. Preparedness and response stage: Strengthening overall preparedness and response capacity is crucial for managing emerging diseases. This includes developing and maintaining emergency response plans, establishing rapid response teams, improving healthcare infrastructure, and enhancing communication and coordination between various stakeholders, including public health agencies, healthcare providers, and communities. It's important to note that prevention strategies should be comprehensive and multifaceted, addressing both human and animal health aspects. Collaboration among different sectors, including human health, animal health, and environmental agencies, is essential for a One Health approach to disease prevention and surveillance. By intervening at these stages and implementing appropriate preventive measures, it is possible to reduce the likelihood of disease emergence and minimize the impact of outbreaks on human health and global well-being.
Prompt 7: What prevention measures do you believe are necessary to prevent the emergence of future pandemics?	Response 7: Preventing the emergence of future pandemics requires a comprehensive and multi-faceted approach. Here are several prevention measures that are crucial in mitigating the risks: Strengthening surveillance systems: Developing robust global surveillance systems to detect and monitor infectious diseases is vital. This includes enhanced early warning systems, real-time data sharing, and improved coordination between countries and international organizations. Promoting wildlife and ecosystem protection: Recognizing the close relationship between human health and the health of ecosystems is important. Measures such as protecting natural habitats, regulating wildlife trade, and promoting sustainable practices can help reduce the risk of zoonotic spillover events. Enhancing biosecurity measures: Implementing strict biosecurity measures in high-risk areas, such as live animal markets, farms, and healthcare facilities, can reduce the likelihood of pathogen transmission. This includes improved hygiene practices, proper waste management, and safe handling and processing of animals. Improving healthcare infrastructure and capacity: Strengthening healthcare systems, particularly in regions at high risk of disease emergence, is essential. This involves investing in healthcare infrastructure, training healthcare workers, ensuring access to diagnostics, and enhancing healthcare delivery, including telemedicine capabilities. Supporting research and development: Investing in research and development for infectious diseases is crucial. This includes studying the biology and ecology of pathogens, developing diagnostics, therapeutics, and vaccines, and promoting interdisciplinary collaboration to advance our understanding of emerging diseases. Enhancing public awareness and education: Promoting public awareness about infectious diseases, their transmission, and preventive measures is vital. Public education campaigns, dissemination of accurate information, and community engagement can help drive behavioral changes and adoption of preventive practices. Strengthening global cooperation: Collaboration among countries, international organizations, and stakeholders is essential for a coordinated response to emerging diseases. This includes information sharing, joint research efforts, capacity building, and supporting resource-constrained regions in their preparedness and response efforts. Implementing One Health approach: Recognizing the interconnectedness of human health, animal health, and the environment, a One Health approach is crucial. This involves integrating expertise from multiple sectors, including human health, veterinary medicine, wildlife conservation, and environmental sciences, to address the complex factors contributing to disease emergence. By combining these prevention measures, it is possible to reduce the likelihood of future pandemics and effectively respond to emerging infectious diseases. Continued vigilance, investment in public health infrastructure, and global cooperation are vital in mitigating the risks and protecting public health.
Prompt 8: Would you like to suggest more prevention measures to halt the next pandemic?	Response 8: Certainly! Here are a few more prevention measures that can contribute to halting the next pandemic: Strengthening global early warning systems: Investing in advanced disease surveillance technologies, including real-time monitoring of human and animal health data, can help identify and track emerging pathogens. Early detection enables swift response and containment measures. Enhancing vaccine development and distribution: Prioritizing the development and production of vaccines against known high-risk pathogens can help prevent future pandemics. Additionally, ensuring equitable access to vaccines globally is crucial to control the spread of infectious diseases. Investing in antiviral research: Developing antiviral therapies that can effectively treat a broad range of viral infections can be instrumental in managing and mitigating the impact of emerging diseases. Strengthening laboratory capacity: Improving laboratory infrastructure and diagnostic capabilities worldwide enhances the ability to quickly identify and characterize emerging pathogens. This enables early response and appropriate public health measures. Implementing effective travel and border health measures: Developing and implementing robust travel and border health measures, including health screening, quarantine protocols, and risk-based travel restrictions, can help prevent the importation and spread of infectious diseases across borders. Promoting global health security financing: Adequate and sustained investment in global health security is crucial for building resilient healthcare systems, strengthening surveillance networks, and supporting research and response efforts. International collaboration and financial support are essential to address these challenges effectively. Encouraging responsible antibiotic use: Promoting prudent use of antibiotics and combatting antimicrobial resistance can prevent the emergence and spread of drug-resistant infections, reducing the risk of future pandemics. Fostering international cooperation in outbreak response: Strengthening international collaboration frameworks, sharing best practices, and fostering rapid information exchange during outbreaks can facilitate a coordinated and effective response. Investing in pandemic preparedness and response training: Providing training and resources to healthcare workers, public health professionals, and emergency responders in pandemic preparedness and response can enhance readiness and resilience in managing future outbreaks. Conducting research on novel pathogens: Investing in research on emerging and unknown pathogens, including those with pandemic potential, can deepen our understanding of their origins, transmission dynamics, and effective countermeasures. It's important to implement a combination of preventive measures to address the multifaceted nature of emerging diseases. By integrating these strategies, we can enhance our ability to prevent, detect, and respond to future pandemics effectively.
Prompt 9: What is your conclusion?	Response 9: In conclusion, preventing the emergence of future pandemics requires a comprehensive and multi-faceted approach. Early detection, surveillance, and monitoring of potential pathogens in both animal and human populations are crucial. Promoting wildlife and ecosystem protection, implementing biosecurity measures, and strengthening healthcare infrastructure are essential preventive measures. Investing in research, development, and global cooperation, along with public awareness and education, can further contribute to halting the next pandemic. Equitable access to vaccines, enhancing laboratory capacity, responsible antibiotic use, and effective travel and border health measures are additional key components. By combining these preventive measures and fostering international collaboration, we can enhance our preparedness, response, and overall global health security.

As the conversation ended, it is important to note that guessing the future pandemic is a multidimensional and challenging assignment, and there are limitations to what AI language models like ChatGPT can accomplish. As precise prediction depends on the quality and quantity of the already generated dataset and AI models are completely dependent on these datasets, they may face biases that are incorporated in the data. To encounter this limitation, collaboration among public health experts, clinicians, researchers, and policymakers across the globe is needed to construct good quality and diverse datasets that will mimic a wide range of inhabitants and atmospheres. Protecting the privacy and security of health information is another challenge during global data sharing. In this regard, there is an urgent need to formulate strict guidelines and standard operating procedures to overcome the challenges. Another limitation of the AI model is the probability of false positive and false negative predictions. False positive prediction might create excessive anxiety and overreaction in the community, whereas false negative prediction can create delayed response leading to increased morbidity and mortality. Despite these drawbacks, ChatGPT is an appreciated model that will help in improving our capability to predict and respond to a pandemic.

In conclusion, predicting future pathogens and formulating prevention strategies in advance is crucial for a prompt and effective pandemic response. AI language models like ChatGPT are not a replacement for human knowledge, experience, and interpretation. These models might deliver valuable insights and predictions of potential outbreaks on the basis of published or available data; it is ultimately the job of public health experts and policymakers to make necessary decisions based on these predictions, experiences, and feedback from the researchers. Moreover, the application of this AI tool in the prediction model is yet to be explored further by mapping the dimensions of various challenges.
